# Molecular evidence for the involvement of cotton *GhGLP2*, in enhanced resistance to *Verticillium* and *Fusarium* Wilts and oxidative stress

**DOI:** 10.1038/s41598-020-68943-x

**Published:** 2020-07-27

**Authors:** Yakun Pei, Yutao Zhu, Yujiao Jia, Xiaoyang Ge, Xiancai Li, Fuguang Li, Yuxia Hou

**Affiliations:** 1 College of Science, China Agricultural University, No. 2 Yuanmingyuan West Road, Beijing, 100193 China; 2 State Key Laboratory of Cotton Biology, Institute of Cotton Research of the Chinese Academy of Agricultural Sciences, Anyang, 455000 China

**Keywords:** Plant sciences, Plant physiology, Plant stress responses

## Abstract

Germin-like proteins (GLPs) are a diverse and ubiquitous family of plant glycoproteins belonging to the cupin super family; they play considerable roles in plant responses against various abiotic and biotic stresses. Here, we provide evidence that GLP2 protein from cotton (*Gossypium hirsutum*) functions in plant defense responses against *Verticillium dahliae*, *Fusarium oxysporum* and oxidative stress. Purified recombinant GhGLP2 exhibits superoxide dismutase (SOD) activity and inhibits spore germination of pathogens. Virus-induced silencing of *GhGLP2* in cotton results in increased susceptibility to pathogens, plants exhibited severe wilt on leaves, enhanced vascular browning and suppressed callose deposition. Transgenic *Arabidopsis* (*Arabidopsis thaliana*) plants overexpressing *GhGLP2* showed significant resistance to *V. dahliae* and *F. oxysporum*, with reduced mycelia growth, increased callose deposition and cell wall lignification at infection sites on leaves. The enhanced tolerance of *GhGLP2*-transgenic *Arabidopsis* to oxidative stress was investigated by methyl viologen and ammonium persulfate treatments, along with increased H_2_O_2_ production. Further, the expression of several defense-related genes (*PDF1.2*, *LOX2*, and *VSP1*) or oxidative stress-related genes (*RbohD*, *RbohF*) was triggered by *GhGLP2*. Thus, our results confirmed the involvement of *GhGLP2* in plant defense response against *Verticillium* and *Fusarium* wilt pathogens and stress conditions.

## Introduction

Cotton (*Gossypium hirsutum* L.) is an important fiber crop that is considered the backbone of the global fiber economy^1^. *Verticillium* and *Fusarium* wilt are caused by *Verticillium dahliae* and *Fusarium oxysporum*, respectively, which are soil-borne pathogenic fungi that present major constraints to the production of cotton^2^. *Verticillium* wilt is a notorious and devastating disease of cotton^3^, and it occurs before the squaring stage and peaks in the boll-setting stage, causing necrotic areas on the leaves, wilting, and discoloration of the vascular tissues^4^. The disease symptoms of *Fusarium* wilt initiate and peak at the seedling and squaring stages, respectively, showing necrotic patches between the main veins and leaf detachment from the stem^5^. Due to diverse factors, such as the climate, pathogen population structures, and cultivar susceptibility, the currently available control measures for these two diseases are not adequate^6^. Thus, research on the cultivation of resistant cotton plants, by finding novel disease-resistant genes against these soil-borne fungal species, is consequently, of great importance.

Previous studies have suggested that extracellular germins and germin-like proteins (GLPs) could be induced by a range of abiotic or biotic stresses, such as herbivores^7,8^, drought^9,10^, salinity^8,11^ and they are considered to be the pathogenesis-related proteins (PRs) 16 family due to their disease resistance property^12–14^. Germins and GLPs were first characterized in wheat (*Triticum aestivum*) and constitute a large plant gene family^15^. They occur as water-soluble glycoproteins, belonging to the cupin superfamily^16^. Most of them are typically hexameric, being trimers of dimers, and are highly resistant to proteases, high temperature, detergents^16,17^.

Recently, evidence has accumulated regarding the basal penetration resistance that is modulated by GLPs in plant–pathogen interactions, explained by the deposition of callose-rich papilla and lignin, at the attempted penetration sites^18,19^. Callose is a polymer of glucose residues joined by 1,3-b-D links, which is deposited between the plasma membrane and the inner face of the primary cell wall. Its accumulation occurs against the attack of some pathogens and forms part of the hypersensitive response^20^. Lignin is an amorphous heteropolymer and primarily deposited in cell walls, it is regarded as a component of the defense response in plants^21^; defense-induced lignification is a conserved basal defense mechanism in the plant immune response against (hemi)biotrophic pathogens in a wild range of plant species^22,23^. The callose deposition and lignification have been used as biochemical markers of activated defense responses^24,25^. In a previous study, Wei et al. have suggested the role of HvOxOLP, which was assumed to be a structural protein that can function as a cofactor for cell wall reinforcements, by the cross-linking of plant cell wall components during the formation of papillae^18^. Also, findings have demonstrated that the OsGLP1 is a cell wall-associated protein that involves in disease resistance^19^.

Reactive oxygen species (ROS) such as singlet oxygen (^1^O_2_), hydroxyl radicals (**·**OH), superoxide anions (O_2_^−^), and hydrogen peroxide (H_2_O_2_) are produced in higher plants and exist in the cell in balance with antioxidant molecules under normal conditions^3^. However, oxidative stress occurs when this balance is disrupted due to excess accumulation of ROS; it results in undesirable oxidative damages that contains metabolism disruption, cellular damage and nucleic acid mutation^26,27^. To maintain growth and productivity, plants have evolved antioxidant defense mechanisms to control the production and elimination of ROS, of which a well-known one is the antioxidant enzyme system. Superoxide dismutase (SOD; EC 1.15.1.1), the first line of defense against ROS^28^, is the major scavenger of O_2_^-^ in this system. It has an effect on restricting the ROS-dependent damage and protecting cells from the oxidative burst by dismutation of O_2_^-^ to H_2_O_2_ and O_2_ and often correlated with increased plant resistance to biotic and abiotic stresses^13^. Some GLPs has been proven to be SODs and against oxidative stress by ROS detoxification^14,29,30^. For instance, researchers have found that both GmGLP10 and HaGLP1 are SODs, their transgenic plants exhibit enhanced tolerance to *Sclerotinia sclerotiorum* infection, by promoting H_2_O_2_ production^31,32^; CchGLP exhibit Mn-SOD activity, its transgenic expression in tobacco provided resistance to geminivirus infection, and silencing of *CchGLP* in pepper (*Capsicum chinense* Jacq.) increased susceptibility to geminivirus single and mixed infections^33,34^.

GLPs also function as signaling molecules inducing a range of defense responses either directly or indirectly^16,30^. *BvGLP1* confers resistance against *Verticillium longisporum* and *Rhizoctonia solani*, with elevated levels of H_2_O_2_, by constitutively triggering the expression of several plant defense-related proteins (PR-1, PR-2, PR-3, PR-4, and PDF-1.2)^35^. OsRGLP1 provides protection against *F. oxysporum* that may involve the direct influence of H_2_O_2_ on salicylic acid (SA) and jasmonic acid (JA) signaling pathways, leading to the activation of defense-related genes^13^.

In the present study, the gene encoding cotton *GLP*, *GhGLP2*, was isolated and expressed in *Escherichia coli*. We demonstrated that purified GhGLP2 exhibits SOD activity and inhibits spore germination of *V. dahliae* and *F. oxysporum*. By generating *GhGLP2*-silenced cotton and overexpressed transgenic *Arabidopsis* plants, we explored the potential of *GhGLP2* in enhancing the resistance to the fungal pathogens and oxidative stress. Furthermore, the characteristic of *GhGLP2* as signaling molecule was proven by the expression of several defense- and oxidative stress-related genes. This study will deepen our understanding of the role of *GLPs* in plant defense responses, and may facilitate the development of cotton, with improved tolerance to biotic and abiotic stresses.

## Results

### Expression of GhGLP2 in response to various conditions

**Figure 1:**
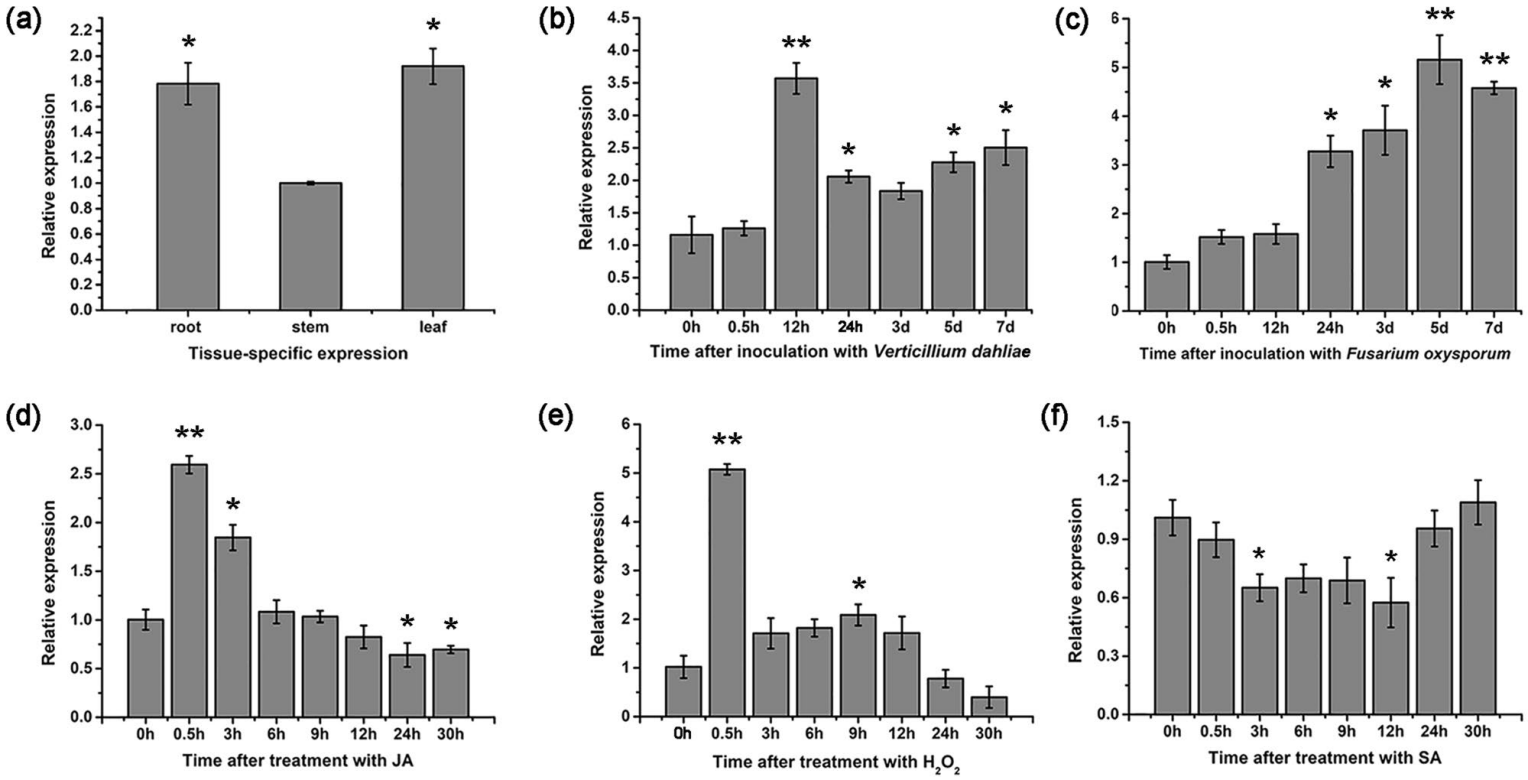
The expression patterns of *GhGLP2* gene under different conditions. (**a**) Tissue-specific expression of *GhGLP2* in cotton. (**b**–**f**) *GhGLP2* expression after inoculation with *Verticillium dahliae* (**b**) and *Fusarium oxysproum* (**c**), and upon treatment with JA (**d**), H_2_O_2_ (**e**), and SA (**f**). *GhGLP2* expression was quantified by qRT-PCR and compared to control. Data were collected from three independent biological samples per treatment and three technical replicates per samples. Error bars represent standard error. Asterisks indicate a significant difference compared with control (*P < 0.05, **P < 0.01, Student’s t-test).

The expression levels of *GhGLP2* under various stress conditions were determined by real-time quantitative reverse transcription-polymerase chain reaction (qRT-PCR) analysis. The tissue-specific expression pattern of *GhGLP2* showed that it was preferentially expressed in leaf, with higher levels observed in the root than in the stem (Fig. 1a). *GhGLP2* expression was significantly induced after pathogen infection; its transcription abundance peaked 12 h and 5 d post inoculation for *V. dahliae* and *F. oxysporum*, respectively (Fig. 1b, c). Upon JA and H_2_O_2_ treatment, *GhGLP2* was immediately up-regulated and reached its maximum expression both at 0.5 h, but dropped to near-basal levels after 3 h and 0.5 h, respectively (Fig. 1d, e). In contrast, in the presence of SA, *GhGLP2* level was slightly decreased at 3 h and 12 h (Fig. 1f).

### Enzymatic and antifungal activity of GhGLP2 protein

The expected recombinant GhGLP2 protein with a molecular weight of 22.7 KDa was expressed in *E. coli* BL21 (DE3), and determined by SDS-PAGE after a 2- to 5-h induction time, with 0.1 mM isopropyl-beta-D-thiogalactopyranoside (Supplementary Fig. S1a). By purifying with Ni columns, purified GhGLP2 was obtained and migrated as doublets on SDS gels (Supplementary Fig. S1b), the difference between the isoforms could be explained by the nature of germin glycan moieties^36^. Matrix-assisted laser desorption/ionization time-of-flight mass spectrometry, was used to further identify the purified protein (Supplementary Fig. S1c); the resultant information was analyzed by a Mascot search (Mascot score 24358; accession no. A0A1U8KA64).

**Figure 2:**
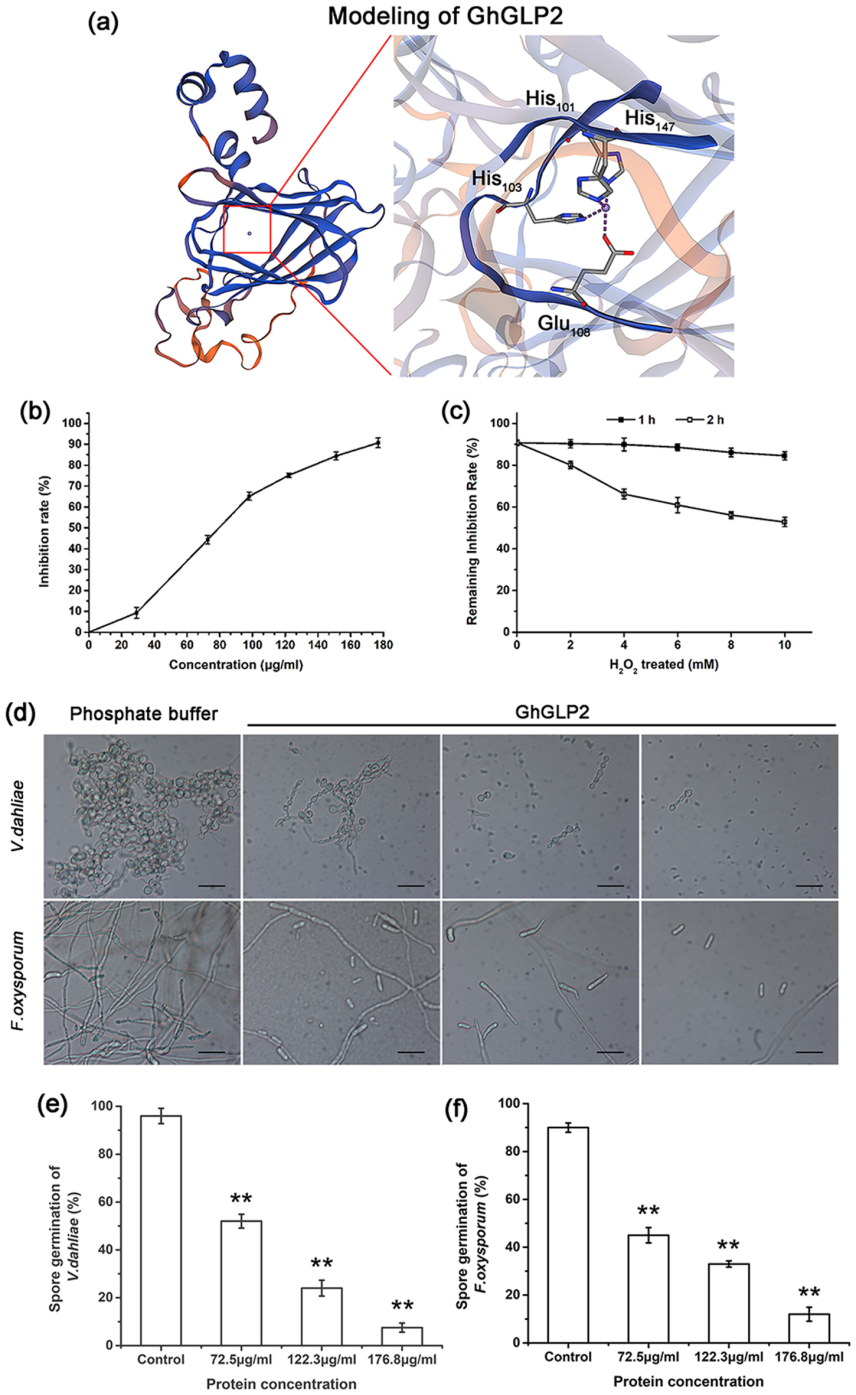
Enzymatic and antifungal activity of GhGLP2. (**a**) Homology modeling of GhGLP2. The highlighted amino acids in GhGLP2 were responsible for protein superoxide dismutase (SOD) activity. (**b**) SOD activity of purified GhGLP2 at concentrations of 29.2, 72.5, 98.0, 122.3, 151.1, 176.8 μg/mL. (**c**) Effect of H_2_O_2_ treatment on the GhGLP2 SOD activity. Protein sample were incubated with H_2_O_2_ concentrations of 0, 2, 4, 6, 8, and 10 mM for 1 and 2 h, respectively. The inhibition rate represents the percentage of SOD remaining activity. (**d**) Spore germination of *Verticillium dahliae* and *Fusarium oxysproum* in phosphate buffer and different concentrations (72.5, 122.3, 176.8 μg/mL) of protein by light microscope at × 40 magnification. (**e**, **f**) Inhibition of spore germination of protein on *V. dahliae* and *F. oxysporum*. Data represent the means ± SD of three independent experiments. Student’s t-test, **P < 0.01 compared to control. Bar = 50 μm.

The 3D model of the GhGLP2 was generated based on the X-ray crystal structure of HvGER (2ET7, with 45.11% similarity), and it was used to identify the active sites responsible for its enzymatic activity. As shown in the GhGLP2 model, three histidine (His101, His103, and His147) and one glutamate (Glu108) residues were responsible for metal ion binding, which is the characteristic of SOD (Fig. 2a). The assay for determining the SOD enzymatic ability of purified GhGLP2 is based on superoxide anion-dependent inhibitory reactions^37^. The SOD activity of GhGLP2 peaked (90.7%) when the protein concentration was 176.8 μg/mL (Fig. 2b). To determine the type of GhGLP2 SOD, purified GhGLP2 protein at a concentration of 176.8 μg/mL was assayed in the presence of H_2_O_2_. The GhGLP2 protein retained stable SOD activity after increasing the concentrations of H_2_O_2_ for 1 h in the reaction mixture (Fig. 2c), indicating that MnSOD was present.

Then, the experiment about inhibition of GhGLP2 protein on the spore germination of *V. dahliae* and *F. oxysporum* was performed. As shown in Fig. 2d, three different concentrations (72.5, 122.3, 176.8 μg/mL) of GhGLP2 protein efficiently caused various degrees of inhibition in terms of spore germination. A total of 96% *V. dahliae* or 90% *F. oxysporum* spores germinated after 30 h of incubation at 25 ℃ and 200 rpm in the control group without ant treatment; at different protein concentrations, spore germination of *V. dahliae* and *F. oxysporum* was significantly reduced within the range of 52.7–7.5% and 45.3–12.5%, respectively (Fig. 2e, f).

### The susceptibility of GhGLP2-silenced cotton plants to *V. dahliae *and *F. oxysporum*

**Figure 3:**
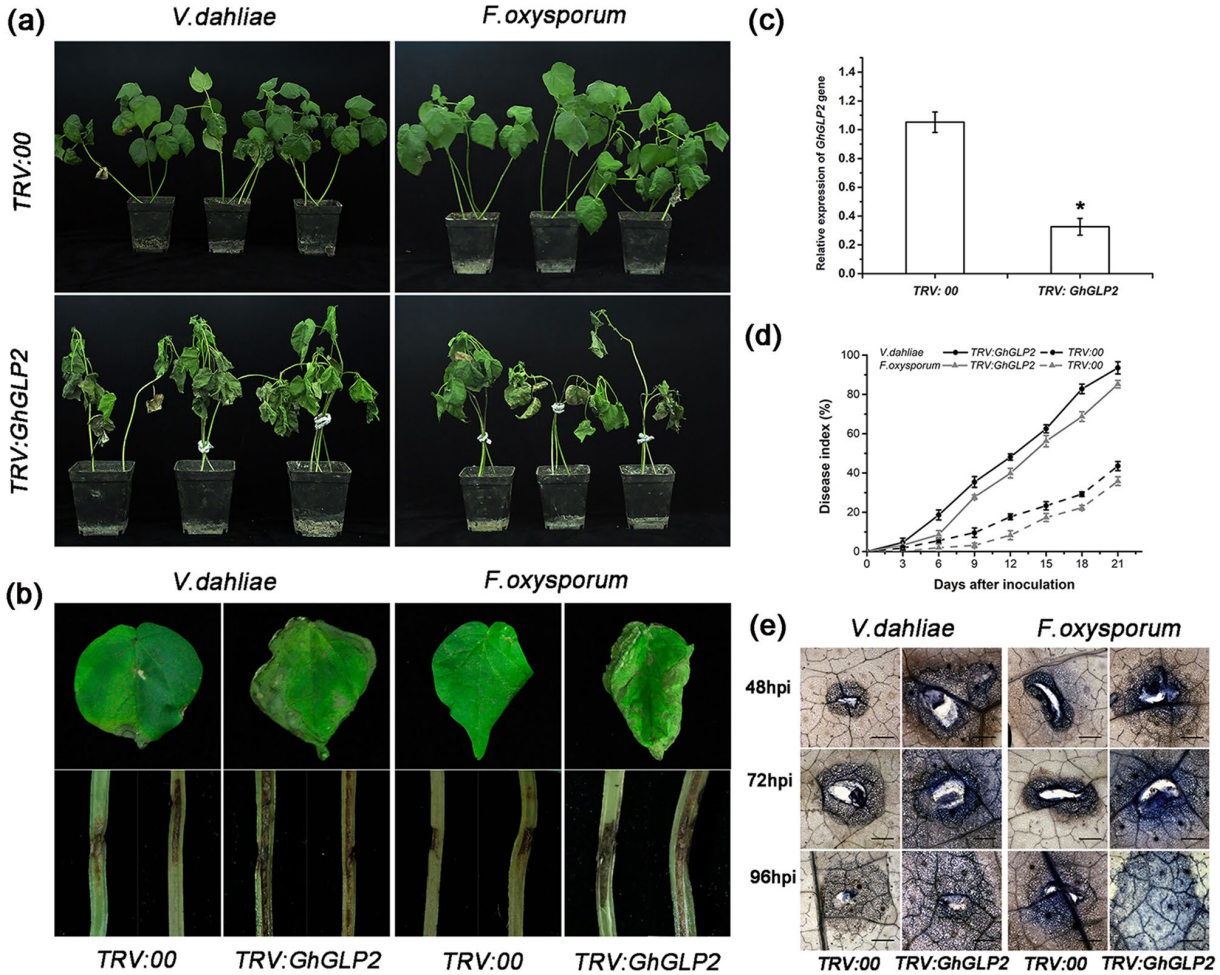
Susceptibility of *GhABP19*-silenced cotton plant. (**a**, **b**) Disease symptoms of *TRV:00* and *TRV:GhGLP2* cotton plants infected by *Verticillium dahliae* and *Fusarium oxysproum* 21 days post inoculation. (**c**) Mean expression levels of *GhGLP2* in VIGS cotton plants. Total RNA was extracted from the leaves of 2-week VIGS cotton. Data represent the means ± SD of three independent biological samples with three technical replicates. Student’s t-test, *P < 0.05, **P < 0.01 compared to control plants. (**d**) Disease index of *TRV:00* and *TRV:GhGLP2* cotton plants at the indicated days after inoculation with *V. dahliae* and *F. oxysporum*, respectively. Error bars represent the standard error of three biological replicates (n ≥ 30). (**e**) Trypan blue staining of *TRV:00* and *TRV:GhGLP2* cotton leaves at 48, 72 and 96 h post-inoculation (hpi) with *V. dahliae* (left panel) and *F. oxysporum* (right panel). The experiments were repeated three times (n ≥ 10).

The *GhGLP2*-silenced cotton plants were generated to examine the functions of *GhGLP2* in disease responses. When the *TRV:GhCLA1* plants showed bleaching in the newly emerged leaves (Supplementary Fig. S3), the lower expression of *GhGLP2* was confirmed by qRT-PCR in the *GhGLP2*-silenced cotton plants (Fig. 3c). Then, both control and *TRV:GhGLP2* cotton plants were inoculated by stem infection. The typical *TRV:GhGLP2* plants exhibited the wilting phenotype in the leaves, developed deeper and larger disease lesions in the stems than those in the control plants, at 21 days post inoculation (dpi) (Fig. 3a, b); parallel results, the percent disease index (PDI) of *TRV:GhGLP2* plants was much lower compared to control plants at different time points after inoculation with *V. dahliae* and *F. oxysporum*, respectively (Fig. 3d). Additionally, an increased area of blue dots was seen in the second true leaves of the infected *GhGLP2*-silenced cotton, which was significantly more than that observed on the leaves of the control plants, at 48, 72, and 96 h post inoculation (hpi), respectively, as determined by trypan blue staining (Fig. 3e).

**Figure 4:**
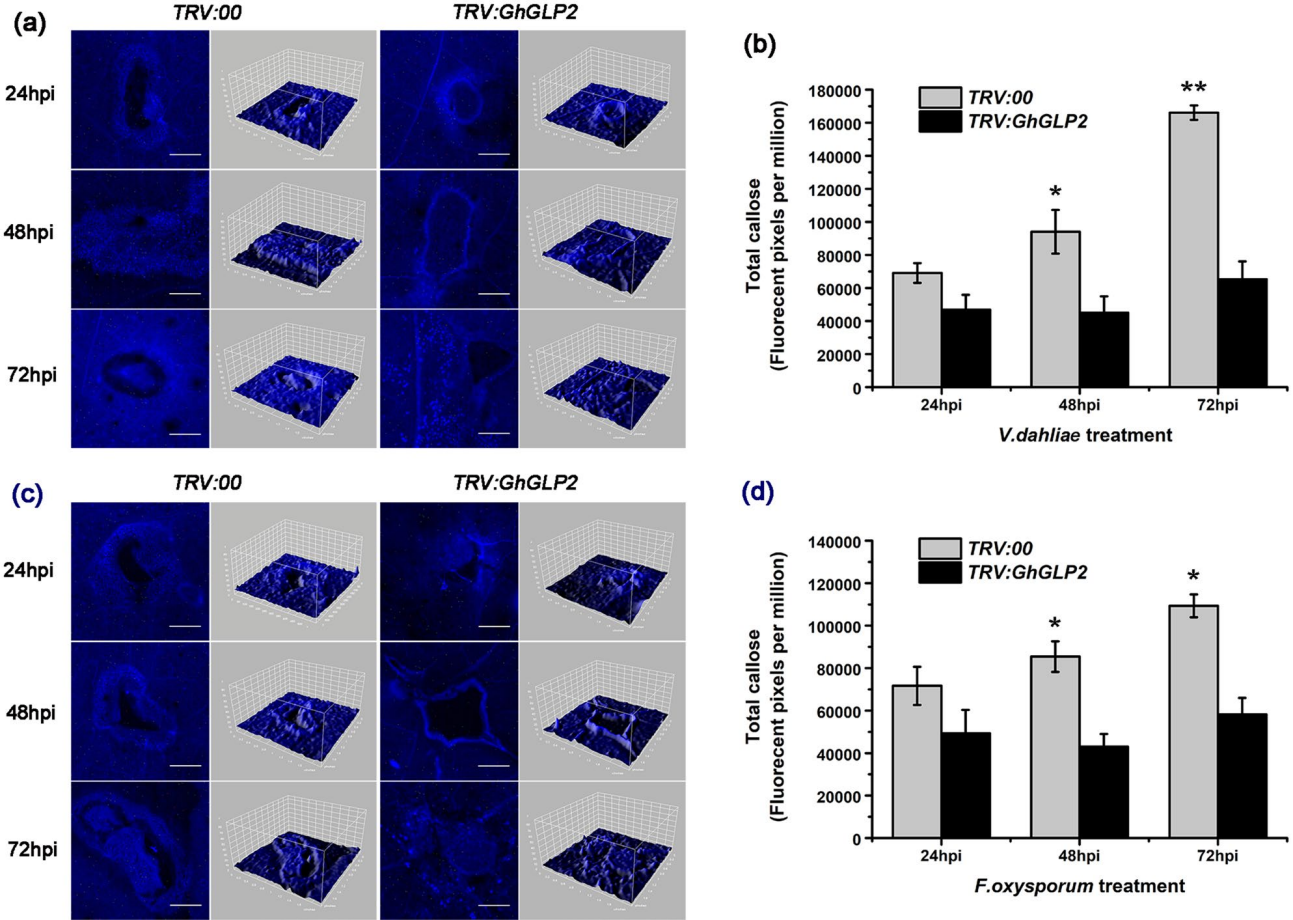
Pathogen-induced callose deposition in *TRV:00* and *TRV:GhGLP2* cotton plants. (**a**, **c**) Representative photographs and 3D surface plots of callose staining in *TRV:00* and *TRV:GhGLP2* cotton plants after inoculation with *Verticillium dahliae* or *Fusarium oxysporum* at 24, 48 and 72 h post-inoculation (hpi). (**b**, **d**) Quantification of total callose in *V. dahliae* or *F. oxysporum* inoculated cotton plants. Data represent the means ± SD of three independent biological replicates (n ≥ 10). Student’s t-test, *P < 0.05, **P < 0.01 compared to wild-type or control plants. Bar = 500 μm.

Aniline blue staining of *V. dahliae*/*F. oxysporum*-infected cotton plants showed that pathogen-induced callose depositions in *GhGLP2*-silenced cotton did not exceed that observed in the controls (Fig. 4). In detail, callose depositions in the *TRV:00* plants presented as a layer in the border of the infection site instead of the dot-shaped or circular deposits in the *TRV:GhGLP2* plants at 48 and 72 hpi; the fluorescence intensity was visualized in 3D surface plots (Fig. 4a, c). Quantification of pathogen-induced callose depositions was determined by the relative number of fluorescence pixels on the digital photographs; it showed a general increasing trend in the *TRV:00* plants from 48 to 72 hpi, but little different was exhibited in *TRV:GhGLP2* plants at different time points (Fig. 4b, d).

### The resistance of GhGLP2-transgenic Arabidopsis plants to *V. dahliae *and* F. oxysporum*

**Figure 5:**
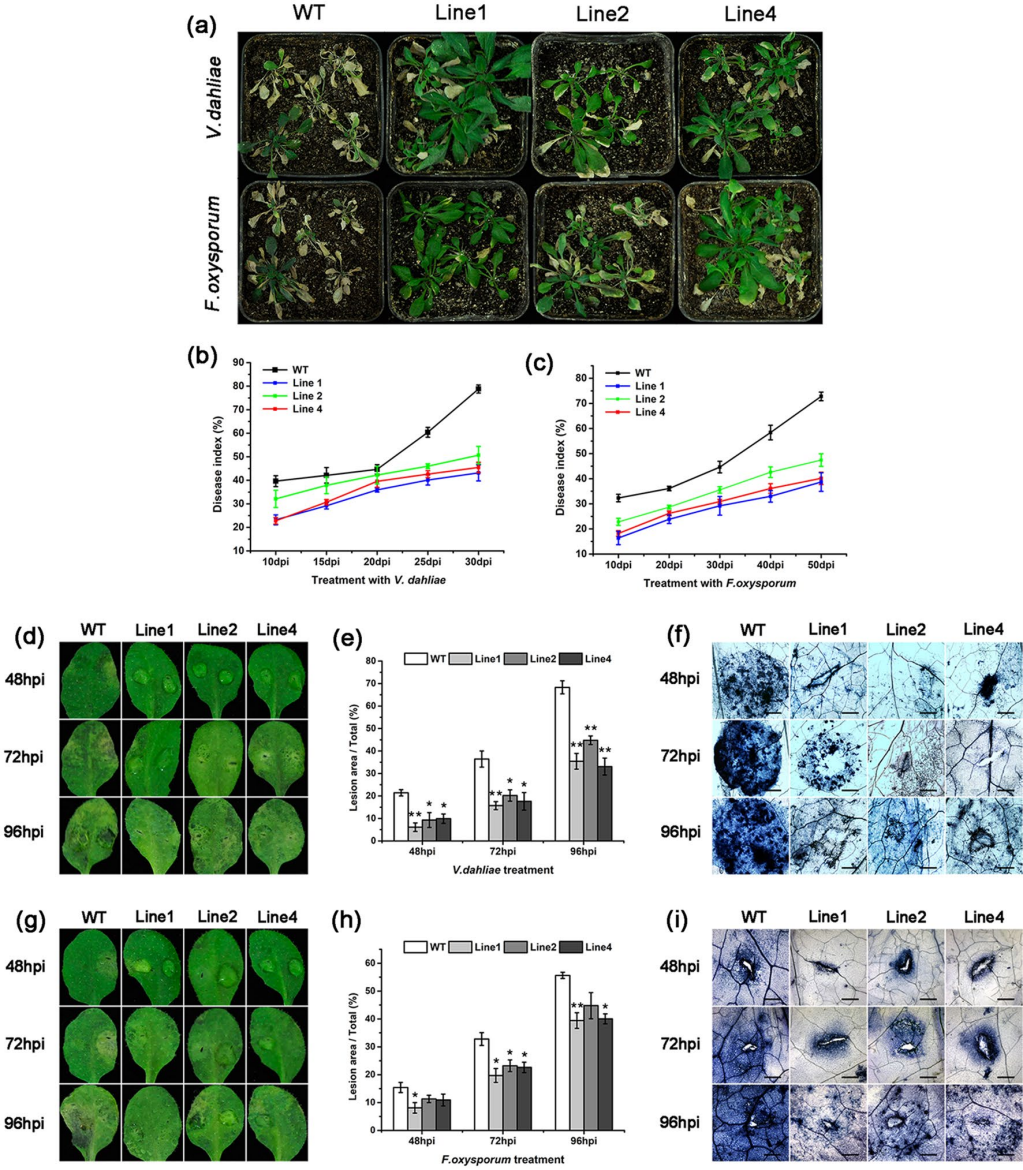
The resistance of wild-type (WT) and *GhGLP2*-transgenic *Arabidopsis* plants. (**a**) Disease symptoms of *Arabidopsis* plants infected with *Verticillium dahliae* or *Fusarium oxysporum* in soil 30 or 50 days post inoculation (dpi), respectively. (**b**, **c**) Plant disease indexes of *Arabidopsis* plants after inoculation with *V. dahliae* (**b**) and *F. oxysporum* (**c**). (**d**–**i**) Disease symptoms (**d, g**), lesion areas (**e, h**) and trypan blue staining (**f, i**) on detached leaves of *Arabidopsis* plants after inoculation with *V*. *dahliae* (**d**–**f**) or *F*. *oxysporum* (**g**–**i**) at 48, 72 and 96 h post-inoculation (hpi), respectively. Data represent the means ± SD of three independent biological replicates (n ≥ 10). Student’s t-test, *P < 0.05, **P < 0.01 compared to wild-type plants. Bar = 500 μm.

*GhGLP2* was ectopically overexpressed in *Arabidopsis* to investigate its resistance effects. Transgenic *Arabidopsis* lines 1, 2, and 4 were selected for subsequent experiments due to their high expression levels (Supplementary Fig. S2b). The roots of the wild-type and *GhGLP2*-transgenic *Arabidopsis* lines were inoculated with suspensions of *V. dahliae* and *F. oxysporum* conidia, to analyze the disease symptoms. Notably, compared with those in the wild-type, the leaf symptoms of wilting, yellowish color, and necrosis, were considerable reduced, and PDI was significantly decreased in the transgenic *Arabidopsis* plants at 30 or 50 dpi, when inoculated with *V. dahliae* or *F. oxysporum*, respectively (Fig. 5a–c). The detached leaves of *Arabidopsis* were drop inoculated with pathogens, and the lesion areas were smaller in the transgenic *Arabidopsis* lines than that in the wild-type plants, at 48, 72, and 96 hpi; (Fig. 5d, e, g, h). In addition, the results of trypan blue staining of the infected leaves indicated that the number of dead cells increased faster in the wild-type than in the transgenic plants, with larger and expanding lesion areas observed beyond the inoculation sites at various time points (Fig. 5f, i). As presented in our study, the germination and growth of *V. dahliae* and *F. oxysporum* proliferated more quickly in the wild-type plants, and were significantly more attenuated in the transgenic plants.

**Figure 6:**
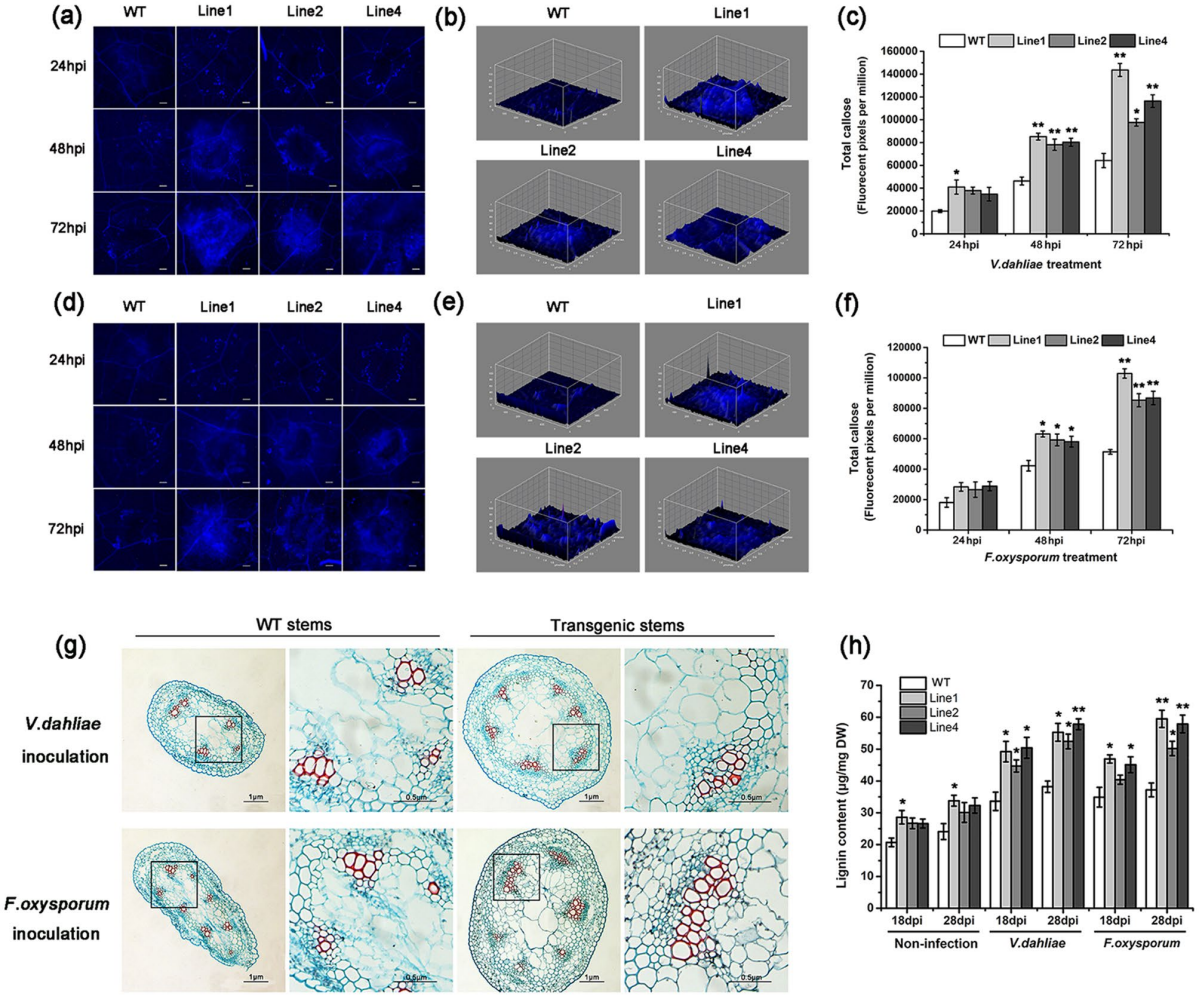
Pathogen-induced callose deposition and lignification in wild-type (WT) and *GhGLP2-*transgenic *Arabidopsis* plants. (**a**, **d**) Representative photographs of callose staining in WT and *GhGLP2-*transgenic *Arabidopsis* plants after inoculation with *Verticillium dahliae* or *Fusarium oxysporum* at 24, 48 and 72 h post-inoculation (hpi). (**b**,** e**) 3D surface plots of callose deposits at 72 hpi with *V. dahliae* or *F. oxysporum*. (**c, f**) Quantification of total callose in *V. dahliae* or *F. oxysporum* inoculated *Arabidopsis* plants. (**g**) Histochemical detection of lignin by Safranin O and Fast Green on the stem cross-sections of WT (left panel) and *GhGLP2*-transgenic *Arabidopsis* line 1 (right panel) at 30 days post-inoculation (dpi) with *V. dahliae* or *F. oxysporum*. Red section represents lignin stained by Safranin O; green section represents cellulose stained by Fast Green. (**h**) Total lignin content detected in *Arabidopsis* plants under the conditions of non-infection, *V. dahliae* and *F. oxysporum* infection. Data represent the means ± SD of three independent biological replicates (n ≥ 10). Student’s t-test, *P < 0.05, **P < 0.01 compared to wild-type or control plants. Bar = 1 μm.

*GhGLP2*-transgenic *Arabidopsis* infected with *V. dahliae* or *F. oxysporum* showed significantly increased callose deposits compared with inoculated wild-type *Arabidopsis* at 48 and 72 hpi (Fig. 6a–f). The shape of callose deposits changed from an enlarged dot-like pattern at 24 hpi to a patch-like formation at 72 hpi in transgenic *Arabidopsis* leaf tissues, whereas it did not change significantly in the wild-type plants (Fig. 6a, d). The increased fluorescence intensity at 72 hpi was presented as 3D surface plots (Fig. 6b, e). Lignin is a tough, water-repellent plant polymer whose production is stimulated by H_2_O_2_ to add structural strength and provide resistance to biodegradation; thus, protecting plant cells^38^. To detect the effect of *GhGLP2* on the formation of lignin, safranin O-fast green was used to detect lignin in the wild-type, and *GhGLP2*-transgenic *Arabidopsis* line 1 at 30 d after inoculation with *V. dahliae* and *F. oxysporum*. As shown in Fig. 6g, the cross-sections of *GhGLP2*-transgenic *Arabidopsis* stems showed increased lignified xylem bundles, vessels, and interfascicular fibers, compared with those in the wild-type plants. These distinct features prompted the performance of additional analyses to better define the lignin content. Consequently, we detected more lignin content in the inoculated *Arabidopsis* than that in the uninoculated ones and in the *GhGLP2*-transgenic *Arabidopsis* than in the wild-type at both 18 and 28 dpi (Fig. 6h), which indicated that *GhGLP2* may be involved in the formation of lignin in plants.

### The tolerance of GhGLP2-transgenic *Arabidopsis* to oxidative stress

**Figure 7:**
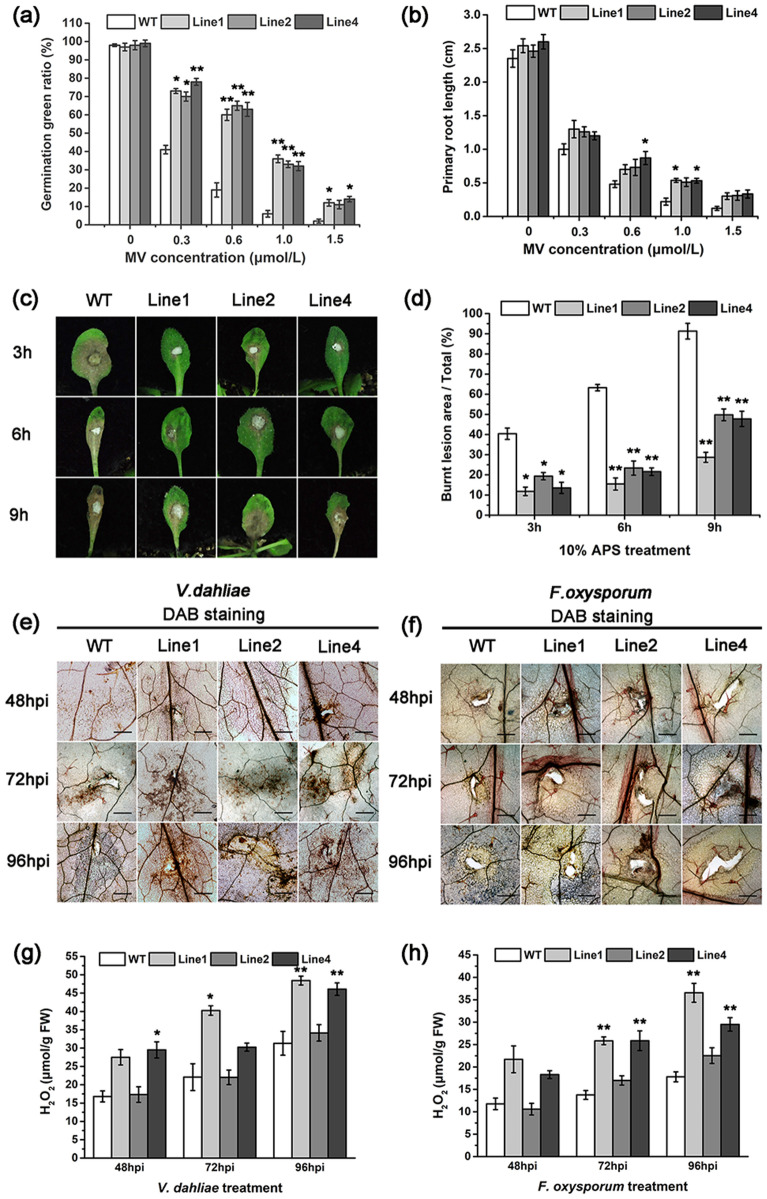
Oxidative stress tolerance and H_2_O_2_ accumulation in wild-type (WT) and *GhGLP2-*transgenic *Arabidopsis* plants. Seedling greening ratios (**a**) and primary root length (**b**) of WT and *GhGLP2-*transgenic *Arabidopsis* plants grown for 10 days on MS medium containing a range of concentrations (0, 0.3, 0.6, 1.0, 1.5 μmol/L) of methyl viologen (MV). (**c**, **d**) Effect of ammonium persulfate (APS) treatment under *in planta* condition. Leaves from uninoculated *Arabidopsis* plants were treated with 10% APS for 3, 6 and 9 h. (**e**–**h**) Histochemical detection of H_2_O_2_ in *Arabidopsis* plants by DAB staining (**e**, **f**) and H_2_O_2_ content analysis (**g**, **h**) at 48, 72 and 96 h post-inoculation (hpi) with *Verticillium dahliae* or *Fusarium oxysporum*. Data represent the means ± SD of three independent biological replicates (n ≥ 10). Student’s t-test, *P < 0.05, **P < 0.01 compared to control plants. Bar = 500 μm.

Based on previous studies that genes harboring SOD activity could circumvent oxidative stress, we investigated the tolerance of *GhGLP2*-transgenic *Arabidopsis* plants to methyl viologen (MV)-mediated oxidative stress. Within the range of MV concentrations (0.3–1.5 μmol/L), germination rate of both wild-type and *GhGLP2*-transgenic *Arabidopsis* declined. Nevertheless, seedling greening ratios of *GhGLP2*-transgenic *Arabidopsis* was higher than that of the wild-type (Fig. 7a). When the concentration of MV were 0.6 and 1.0 μmol/L, the primary root length of *GhGLP2*-transgenic *Arabidopsis* was longer than wild-type after grown for 10 days (Fig. 7b). In addition, the *GhGLP2*-transgenic *Arabidopsis* plants were analyzed for their tolerance to the strong oxidizing agent, ammonium persulfate (APS), under *in planta* conditions. As excepted, the burnt lesions in the transgenic leaves were smaller than those in the wild-type, after 3, 6, and 9 h of APS treatments (Fig. 7c, d).

Furthermore, we determined the effects of overexpressing *GhGLP2* on H_2_O_2_ accumulation in *Arabidopsis* plants. In pathogen-inoculated transgenic *Arabidopsis* plants, more brown spots were observed in and around the infection sites at 48, 72, and 96 hpi, with *V. dahliae* or *F. oxysporum*, indicating the accumulation of H_2_O_2_ (Fig. 7e, f). Consistently, H_2_O_2_ quantitation was found to peak at 96 hpi in infected transgenic *Arabidopsis* lines 1 and 4, which was almost 1.5-fold greater than that in the wild-type plants (Fig. 7g, h).

### The expression of defense- and oxidative stress-related genes

**Figure 8:**
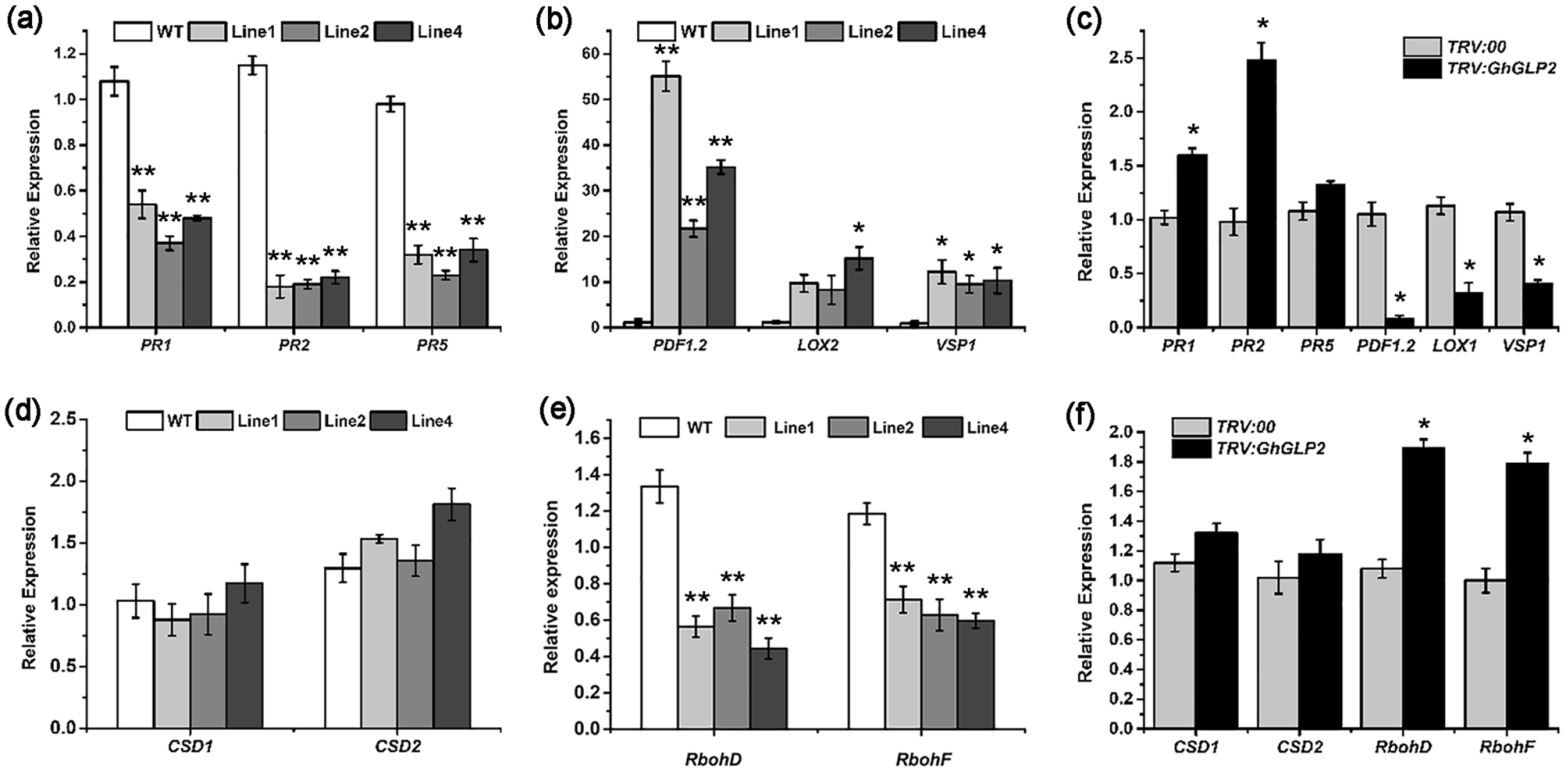
qRT-PCR analysis of defense- and oxidative stress-related genes in uninoculated *GhGLP2*-transgenic *Arabidopsis* and silenced cotton plants. (**a**–**c**) The relative expression of SA- (*PR1*, *PR2*, *PR5*) and JA-dependent (*PDF1.2*, *LOX2/1*, *VSP1*) defense genes in uninoculated *GhGLP2*-transgenic *Arabidopsis* (**a**, **b**) and *GhGLP2*-silenced cotton plants **(c)**. (**d**–**f**) The relative expression levels of ROS scavenging (*CSD1*, *CSD2)* and generating (*RbohD*, *RbohF)* genes in uninoculated *GhGLP2*-transgenic *Arabidopsis* (**d, e**) and *GhGLP2*-silenced cotton plants (**f**). *AtEF1a* and *GhUBQ7* were used as internal standards in *Arabidopsis* and cotton, respectively. Data represent the means ± SD of three independent biological samples with three technical replicates. Student’s t-test, *P < 0.05, **P < 0.01 compared to wild-type or control plants. *WT* wild-type.

To define whether the resistance of *GhGLP2* is correlated with the expression of other defense-related genes, we analyzed several of them in defense-related SA and JA signaling. The results in Fig. 8a showed that the SA-related genes *PR-1*, *PR-2*, and *PR-5* were downregulated in the non-inoculated *GhGLP2*-transgenic *Arabidopsis* by approximately 2-, 5-, and 3-folds, compared with those in the wild-type, respectively; whereas the JA-related genes *PDF1.2* and *VSP1* were significantly upregulated in transgenic *Arabidopsis* lines (Fig. 8b). Contrarily, the expression of *PR-1* and *PR-2* was slightly upregulated, and a strong transcriptional downregulation of *PDF1.2*, *LOX1*, and *VSP1* was observed in the *GhGLP2*-silenced cotton compared with that in the control plants (Fig. 8c).

As the *GhGLP2* overexpression enhances the resistance to oxidative stress in transgenic *Arabidopsis* plants, the expression of several ROS-related genes was evaluated. The genes that encode the ROS-scavenging [*CSD1* (AT1G08830), *CSD2* (AT2G28190)] and -generating enzymes [*RbohD* (AT5G47910), *RbohF* (AT1G64060)] were analyzed by qRT-PCR. The results demonstrated no significant differences in the expression of *CSD1* and *CSD2* between the transgenic *Arabidopsis* lines and wild-type (Fig. 8d); while the *RbohD* and *RbohF* genes were sharply downregulated (Fig. 8e). Moreover, compared with that in the control cotton, no change or slight induction was observed in the expression of ROS-scavenging or -generating genes in the *GhGLP2*-silenced cotton plants (Fig. 8f).

## Discussion

GLPs are known to be involved with disease resistance in a broad range of plants. In this study, the *GhGLP2* gene from cotton was cloned, its enzymatic functions and the defense mechanisms were elucidated through *V. dahliae-* and *F. oxysporum-*infected transgenic plants, which were generated by endogenous gene silencing in cotton plants and heterologous gene expression in *Arabidopsis* plants.

The general structure of GhGLP2 is consistent with the typical organization of GLPs, the most important characteristic they shared is the three motifs, box A (QDFCVAD), box B (G-P-H-HPGASEXXXXX-G) and box C (GXXGFQ-N-G) (Supplementary Fig. S4)^39^. A conserved cysteine residue (Cys-31) was contained in box A, believed to form an internal disulphide bond with a second external cysteine (Cys-40) and to stabilize the protein structure^40^. Box B (GINMPHFHPRATEIAFVLEG) and box C (GLLHFQMNVG) in GhGLP2 contain three highly conserved histidine (His-101, His-103, His-147) and one glutamate (Glu-108) residues that involved in metal ion binding^16,39^. Another conserved tripeptide KGD-like was occurred in GhGLP2 (KGE). In plants, KGD-like motif was reported to be involved in protein–protein interactions, pathogen resistance via plasma membrane cell wall adhesion^41^ and in the prevention of fungal toxin penetration^42^. Since the presence of a N-terminal signal peptide supports the apoplastic localization, GLPs are usually found associated with cell wall. In addition, a phylogenetic tree was generated to examine the phylogenetic relationships of GLPs from various plants, GhGLP2 was clustered in subfamily 1 (Supplementary Fig. S5).

GLPs are essential for plant defense resistance to biotic stress^12^, here, to investigate the possible role of *GhGLP2* in response to pathogen infection, its transcript abundance was analyzed by qRT-PCR. First, we found *GhGLP2* was preferentially expressed in root and leaf tissue, indicating conserved gene regulation under normal growth conditions (Fig. 1a). When cotton plants inoculated with *V. dahliae* and *F. oxysporum*, the expression levels of *GhGLP2* were peaked at 12 hpi and 5 dpi by about 3.5- and fivefold, respectively, suggesting its potential role in the plant basal resistance (Fig. 1b, c).

GLPs are known to be associated with various enzymatic activities^43–47^. In our study, the SOD activity of GhGLP2 was verified by predicting the active sites in the 3D model, and generating in vitro expressed recombined proteins (Fig. 2a–c and Supplementary Fig. S1). In addition, according to the metal ion cofactor required for their activity, SODs can be categorized into three types, including Cu/ZnSOD, FeSOD and MnSOD; in the presence of H_2_O_2_, MnSOD was stable, whereas Cu/ZnSOD and FeSOD are known to lose activity following this treatment^48^. After incubating with various concentrations of H_2_O_2_ for 1 h, the SOD activity of GhGLP2 was remains, indicting its MnSOD property (Fig. 2c).

GLPs with SOD activity from wheat and barley have long been known to provide resistance against *Blumeria graminis*^49^. Recently, more defense-related GLP SODs have been found in heterologous systems such as rice^13^, pepper^34^, sunflower^31^, and rape^50^. In the present study, we obtained that GhGLP2 protein significantly restricted spore germination of the tested *V. dahliae* and *F. oxysporum* in a dose-dependent manner (Fig. 2d–f). The protein exhibited antifungal activity that might be attribution to the presence of SOD activity. Then, the *GhGLP2*-silenced cotton plants and *GhGLP2*-transgenic *Arabidopsis* plants were generated to determine the role of *GhGLP2* in pathogen resistance. By decreasing the expression of *GhGLP2* in cotton plants, the *GhGLP2*-silenced cotton was more susceptible to *V. dahliae* and *F. oxysporum* infection, as evidenced by the greater severity of the disease symptoms, such as deepened vascular browning, large lesion sizes, and higher PDI compared with those in the control plants (Fig. 3). The overexpression of *GhGLP2* in *Arabidopsis* interferes with *V. dahliae* and *F. oxysporum* root infections and provides substantive resistance (Fig. 5a-c); it also limits the virulence and growth of the fungi on the detached leaves of *Arabidopsis* plants, as observed by trypan blue staining (Fig. 5f, i). Apparently, the symptoms of the *GhGLP2*-overexpressed *Arabidopsis* plants were restricted to the inoculation sites, indicating that *GhGLP2* might determine the ability of fungal pathogens to degrade the plant tissues.

Previous literature has shown that several GLPs are targeted to the extracellular space and postulated to have structural roles in relation to the cross-linking of cell walls after fungal pathogen attacks^49,51,52^. Callose deposited between the plasma membrane and cell wall and lignin primarily deposited in cell walls, they act as a physical barrier to stop or slow the invading pathogens^53,54^ and lignification of cell walls is a key event in the resistance against fungal penetration^24^. In the present study, reduced callose production was observed in *GhGLP2*-silenced cotton plants challenged with *V. dahliae* or *F. oxysporum*, as evidenced by decreased fluorescence intensity (Fig. 4). Furthermore, as expected, callose accumulation was enhanced in transgenic *Arabidopsis* (Fig. 6a–f). Here, lignification was clearly observed, with the appearance of larger red areas in the cross-sections of infected transgenic *Arabidopsis* plants (Fig. 6g). Moreover, by measuring the quantitative content of total lignin, increased lignin accumulation was detected in the inoculated *Arabidopsis* (Fig. 6h). Additionally, the GhGLP2:GFP protein was found to be expressed in the cell wall after plasmolysis (Supplementary Fig. S6). Thus, it can be deduced that subcellular localization of GhGLP2 provides possibilities for callose formation and cell wall lignification, which contributes to the reinforcement of cell wall, to protect cells from pathogen penetration.

Oxidative stress can arise from an imbalance between generation and elimination of ROS, resulting in excessive ROS levels causing damage to almost all biomolecules, leading to cell death^55^. MV is a known oxidative stress inducer^56^, the antioxidant defenses mediated by GhGLP2 SOD were investigated in seedlings of *GhGLP2*-transgenic *Arabidopsis* plants. We found that transgenic *Arabidopsis* showed heightened tolerance to MV-mediated oxidative stress as compared to the wild-type with high germination rate and long primary root (Fig. 7a, b). The other strong oxidizing agent APS can induce high accumulation of ROS, which disrupts the photosynthetic processes, by causing the peroxidation of membrane lipids^57^. Our study documented that *GhGLP2*-overexpressed *Arabidopsis* leaves were more tolerant to APS toxicity than the untransformed plant leaves (Fig. 7c, d). Therefore, since GhGLP2 with SOD activity confers resistance to oxidative stress, this suggests that GhGLP2 might protect plants from oxidative damage. To further confirm the antioxidant properties of GhGLP2, microscopically generated H_2_O_2_ in *Arabidopsis* plants were detected by DAB staining methods. Compared to wild-type plants, *GhGLP2*-transgenic *Arabidopsis* showed relatively increase of H_2_O_2_ accumulation when inoculated with *V. dahliae* and *F. oxysporum*, expounded by the overexpression of GhGLP2 SOD, as it eliminates excess ROS and contributes to the H_2_O_2_ levels (Fig. 7e-h).

Furthermore, GLPs act as signaling molecules to activate the expression of host defense response-related genes^31,35,52^. SA and JA/ET have been shown to be important phytohormones involved in the defense responses to pathogen attacks in several plant–pathogen interactions^58^. The *GhGLP2* expression was elevated or slightly suppressed after treatment with exogenous JA or SA (Fig. 1d, f). In this respect, we can speculate that *GhGLP2* likely involved in phytohormone signaling regulation. Then, the transcript levels of SA (*PR1*, *PR2* and *PR5*) and the JA/ET pathway maker genes (*PDF1.2*, *LOX2*, and *VSP1*) were analyzed to identify the involved defense pathways (Fig. 8a–c). The results showed that the SA-dependent genes were significantly downregulated in the transgenic *Arabidopsis* lines. Different from the SA-dependent genes, the *PDF1.2*, *LOX2*, and *VSP1* genes in the JA/ET pathway were upregulated in the transgenic *Arabidopsis* lines. Due to the activation of the JA/ET pathways, the respective genes in the transgenic *Arabidopsis* lines, occurred even in the absence of pathogens, as the plants appear to be better protected. In addition, as shown in Fig. 8c, the *GhGLP2*-deficient cotton plants exhibited enhanced transcript levels of the SA-pathway-related genes *PR1* and *PR2*, but significantly decreased levels of the JA/ET-pathway-related genes *PDF1.2*, *LOX2*, and *VSP1*. In this respect, we can speculate that *GhGLP2* may be functional as a signaling component of plant resistance mechanisms, by specifically regulating the expression of a set of plant defense-related genes prior to the pathogen attack.

We also tested the expression levels of several oxidative stress-related genes (Fig. 8d–f). *CSD1* and *CSD2* (Cu/Zn SOD enzyme genes) are responsible for ROS scavenging, and they are implicated in H_2_O_2_ detoxification and stress responses^59^; *RbohD* and *RbohF* (NADPH oxidase genes) are the most important O_2_^-^ producers during oxidative bursts^60,61^. In transgenic *Arabidopsis* plants, the expression of *CSD1* and *CSD2* was not induced, whereas the two NADPH oxidases showed significant patterns of downregulation. While non-induced *CSD1* and *CSD2* indicated that they did not contribute to H_2_O_2_ production, and the reduced expression levels of *RbohD* and *RbohF* might decrease the formation of O_2_^-^, and increased production of H_2_O_2_ was found in the *GhGLP2*-overexpressd *Arabidopsis* lines, which suggests that H_2_O_2_ accumulation might be a direct consequence of GhGLP2. The overexpression of *GhGLP2* could potentially suppress the expression of ROS-generating genes. Moreover, in agreement with our predictions, the NADPH oxidases were activated by the silencing of *GhGLP2* in cotton.

In conclusion, our study reveals the potential roles of GhGLP2 in plant defense and oxidative stress resistance. By investigating the physiological properties of the inoculated *GhGLP2*-silenced cotton plants and *GhGLP2*-overexpressed *Arabidopsis* plants, we have demonstrated that GhGLP2 promotes efficient defense responses, which results in cell wall reinforcement by callose depositions and lignification in the infection sites. Moreover, GhGLP2 enhances the tolerance to oxidative stress in transgenic *Arabidopsis* plants by its SOD activity. These results provide a foundation for developing new strategies to improve *Verticillium*/*Fusarium* wilt and oxidative stress resistance in cotton plants.

## Methods

### Plant materials and fungal cultivation

Upland cotton (*Gossypium hirsutum* L.) cultivar Zhongzhimian 2 and *Arabidopsis thaliana* (ecotype Columbia) plants were grown on soil in growth chamber under the photoperiod of 16/8 h (light/dark) at 25/22 ℃ and 22/18 ℃, respectively. *Verticillium dahliae* strain VD 991 and *Fusarium oxysporum* were cultured on potato dextrose agar at 25 °C for a week, and colonies were then transferred to Czapek’s liquid medium. Their spore suspensions (10^7^ conidia/mL) were used for inoculation assays.

### Gene expression analysis using qRT-PCR

By screening the transcriptom sequencing of *G. hirsutum* inoculated with *V. dahliae* (PRJNA408075; SRA Database), a germin-like protein gene (KJB72110.1) was found to be a highly up-regulated differential expression gene, which named *GhGLP2* (GenBank accession number: MH430584). To analysis the expression patterns of *GhGLP2*, 2-week-old cotton seedlings were inoculated with *V. dahliae* or *F. oxysporum* conidial suspension on roots for pathogen treatment; true leaves were sprayed with 100 μM MeJA, 100 mM H_2_O_2_, and 1 mM SA for stress treatments, respectively. EASYspin RNA Extraction Kit (Biomed, China) and TRNzol RNA kit [TIANGEN BIOTECH (Beijing) CO., LTD.] were used to isolated total RNA from cotton and *Arabidopsis* leaves, respectively. The cDNAs were synthesized by Fast Quant cDNA Reverse Kit (TIANGEN BIOTECH CO., LTD). qRT-PCR was prepared using SYBR Premix Ex Taq (Tli RNaseH Plus; Takara, Shiga, Japan) in a final volume of 20 μL and performed with ABI 7500 thermocycler (Applied Biosystems, Foster city, CA, USA). The cotton endogenous gene *GhUBQ7* (DQ116441) and *Arabidopsis* housekeeping gene *elongation factor 1a AtEF1a* (AT5G60390) were used as internal standards. Expression was determined by the 2^-ΔΔCT^ method and all primers used for this analysis are shown in Supplementary Table S1. All experiments were carried out with three biological replicates and three technical replicates.

### In vitro expression and enzymatic activity measurement of GhGLP2

The nucleotide sequence encoding GhGLP2 without signal peptide was amplified and cloned into pET-22b vector (Novagen, Madison WI, USA) (primers see Supplementary Table S1).The expression construct was then transformed into *E*. *coli* strain BL21 (DE3) as described previously^37^. After inducing by 1 mM isopropyl-beta-D-thiogalactopyranoside at 37 °C for 4 h with oscillation, cells in 100 mL of Luria Bertani broth were harvested by centrifugation for 20 min at 10,000 × *g* and sonicated for 6 × 30 s at 4 °C in chilled 1 × PBS (pH 7.2). Soluble expression was verified by SDS-PAGE, GhGLP2 was purified using 6 × His-Tagged Protein Purification Kit (CW BIO). Total SOD activity of purified protein and endogenous SOD activity of samples were measured by detection kit according to the manual (Beyotime Institute of Biotechnology, China). To determine the type of purified protein associated SOD activity, the samples were treated with 0, 2, 4, 6, 8 and 10 mM H_2_O_2_ for 1 and 2 h prior to SOD assay.

### Antifungal activity of GhGLP2

Conidial suspensions (10^6^ conidia/mL) of *V. dahliae* and *F. oxysporum* were used in this assay. Phosphate buffer (control; 0.5 ml) or GhGLP2 protein solutions (0.5 ml) with different concentrations (72.5, 122.3, 176.8 μg/ml) were inoculated with a spore suspension (0.5 ml). Then the mixed system was incubated at 25 °C for 30 h in an incubator shaker (200 rpm). Spore germination of *V. dahliae* and *F. oxysporum* was photographed under a Nikon eclipse Ti microscope; the percentage spore germination was calculated by using a haemocytometer.

### Generation of transgenic *Arabidopsis* plants

*GhGLP*2 was amplified with restriction sites *Xba* I and *Spe* I on forward and reverse primers, respectively (primers see Supplementary Table S1). The amplified gene was subcloned into Super-pCAMBIA1300 vector with green fluorescent protein gene (*GFP*) under control of the CaMV35S promoter (Supplementary Fig. S2a). Then the constructed vector was introduced into *Agrobacterium tumefaciens* (strain GV3101) by freeze–thaw method^62^. For *Arabidopsis* transformation, flowering wild-type plants were dipped into *A. tumefaciens*-containing infiltration medium^63^. By observing at 488 nm with FLUOVIEW FV1000 Confocal Laser Scanning Microscopy (OLYMPUS, Tokyo, Japan), the intracellular localization of GhGLP2:GFP fusion protein was determined in the roots of 7-day-old transgenic seedlings. Plasmolysis was induced by incubating samples in 0.8 M mannitol for 10 min.

### Virus-induced gene silencing (VIGS) in cotton plants

A 438 bp fragment of *GhGLP2* was amplified with VIGS-GhGLP2 F/R primers (Supplementary Table S1) and inserted into the TRV:00 vector to construct TRV:GhGLP2. The cloroplastos alterados gene *GhCLA1* was used as a visual marker to determine the efficiency of the VIGS by showing an albino phenotype^1^. TRV:GhCLA1 was constructed according to previous report to detect the efficiency of silencing under our experimental conditions^1^. The plasmids of TRV1 (pYL192), TRV:GhGLP2, and TRV:GhCLA1 were transformed into *A. tumefaciens* strain GV3101 by heat shock. The cultures of *A. tumefaciens* were infected into the cotyledons of 2-weeks-old cotton seedlings as described previously^1^. Cotton treated with equal amounts of *TRV1* and *TRV:00*, *TRV1* and *TRV:GhGLP2*, *TRV1* and *TRV:GhCLA1* were severed as control (*TRV:00*), *GhGLP2*- (*TRV:GhGLP2*) and *GhCLA1*-silenced (*TRV:GhCLA1*) plants, respectively. The silenced efficiency of *GhGLP2* was determined by qRT-PCR. *GhUBQ7* from cotton was amplified as an internal control.

### Inoculation of *V. dahliae* and* F. oxysporum*

For root inoculation of *Arabidopsis*, four-week-old transgenic and wild-type *Arabidopsis* seedlings were removed from the soil and immersed into the spore suspension for 5 min, then immediately replanted into fresh soil^64^. The PDI was calculated as the severity of infection on the plants^37^. For VIGS cotton plants, they were challenged by syringe inoculation as previously reported^1^.

For living plants leaf inoculation, *Arabidopsis* and cotton plants were inoculated with two drops (10 μL) of *V. dahliae* or *F. oxysporum* conidial suspension per leaf (four leaves per plant in *Arabidopsis* and on the true leaves of cotton). In addition, detached leaves were challenged with *V. dahliae* and *F. oxysporum* filtrate by in vitro inoculation. Briefly, *Arabidopsis* or cotton leaves were cut from soil-grown plants and placed adaxial side up on moist filter paper in Petri dishes and 10 μL spore suspension of *V. dahliae* or *F. oxysporum* was spotted at two sites per leaf. Infected leaves were incubated at 25 °C in a dark chamber^65^.

### Cell death and callose deposition in leaves

Trypan blue staining was performed as previously described to view cell death^66^. Briefly, detached leaves were stained with trypan blue in lactophenol solution (10 mL lactic acid, 10 mL glycerol, 10 g phenol, and 40 mg trypan blue dissolved in 10 mL distilled water) by boiling. The stained leaves were immersed in choral hydrate solution (250% w/v) to remove chlorophyll and observed with a Nikon digital camera. Callose deposition was determined by aniline blue staining^67^. Samples were obtained in living plants as described previously. Control leaves were not infected. The quantification of infected areas was performed by calculating the number of fluorescent pixels per million pixels in digital image with ImageJ software.

### H_2_O_2_ and lignin quantification

Inoculated leaves were collected at 48, 72 and 96 hpi and were immersed in DAB-HCl solution for 6–7 h to view H_2_O_2_^68^. Control leaves were not infected. Subsequently, samples were boiled in lactic acid/glycerol/ethanol solution (1:1:3, v/v/v) to remove chlorophyll or unbound stains. The detained leaves were stored in 50% glycerol or visualized with a microscope under bright light. The H_2_O_2_ content was measured with FOX reagent as described previously^69^. For lignin detection, 25 mm stem cross-sections of inoculated wild-type and *GhGLP2*-transgenic *Arabidopsis* were cut on a sliding microtome, fixed in FAA solution, and stained by safranin O-fast green^70^. Microslides were observed with an optical microscope (Nikon ECLIPSE Ti, Tokyo, Japan). Total soluble lignin content was quantified using thioglycollic acid technique^71^.

### MV and APS treatment

To assess the MV tolerance, seeds form wild-type and transgenic *Arabidopsis* lines were sown on MS medium plates without or with a range of MV concentrations (0.3, 0.6, 1.0, 1.5 μmol/L), incubated at 4 ℃ for 3 days, and grown vertically at standard condition. After grown for 7 or 10 days, germination rate or primary root length were measured. The leaves of wild-type and transgenic *Arabidopsis* lines were analyzed for tolerance against APS under *in planta* condition. 20 μL of APS (10%) solution was spotted onto the leaves of *Arabidopsis* plants. The phenotype was recorded after 3, 6 and 9 h of treatment and the burnt lesions (whitish color) / Total areas were measured for knowing the effect of APS on leaves.

### Homology modeling

Homology model of GhGLP2 was generated using SWISS-MODEL (https://www.swissmodel.expasy.org/)72. *Hordeum vulgare* germin protein (2ET7) was used as template. All three-dimensional (3D) models were analyzed and visualized using EzMol 1.3. (https://www.sbg.bio.ic.ac.uk/~ezmol/)73.

### Statistical analysis

All the experiments were repeated three times with a minimum of 10 plants per genotype/condition. Data are shown as the means of the independent biological replicates ± standard deviation (SD). Analysis of variance was carried out with software IBM SPSS statistics 25. Significant differences were determined at the 5 and 1% level of significance and asterisks are used to indicate p-values (*P < 0.05, **P < 0.01).

## Supplementary information

Supplementary information

## Data Availability

All data generated or analysed during this study are included in this published article (and its Supplementary Information files).
