# Supplementary material for: Molecular evidence for the involvement of cotton GhGLP2, in enhanced resistance to Verticillium and Fusarium Wilts and oxidative stress
Source: Sci Rep. 2020 Jul 27;10:12510. doi: 10.1038/s41598-020-68943-x (PMC7385154; doi:10.1038/s41598-020-68943-x)
Supplement: Supplementary file 1 — Supplementary information [file 41598_2020_68943_MOESM1_ESM.pdf]

Molecular evidence for the involvement of cotton *GhGLP2*, in enhanced resistance to *Verticillium* and *Fusarium* Wilts and oxidative stress

Yakun Pei [1], Yutao Zhu [1], Yujiao Jia [1], Xiaoyang Ge [2], Xiancai Li [1], Fuguang Li [2]*, and Yuxia Hou [1]*

[1] College of Science, China Agricultural University, No. 2 Yuanmingyuan West Road, Beijing 100193, China
[2] State Key Laboratory of Cotton Biology, Institute of Cotton Research of the Chinese Academy of Agricultural Sciences, Anyang 455000, China

Yakun Pei: ykP1992@163.com;
Yutao Zhu: zyt17494@163.com;
Yujiao Jia: Jujcau@163.com;
Xiaoyang Ge: gexiaoyang613@163.com;
Xiancai Li: lixiancai312@cau.edu.cn;
Yuxia Hou*: yuxiacau@163.com;
Fuguang Li*: aylifug@126.com.

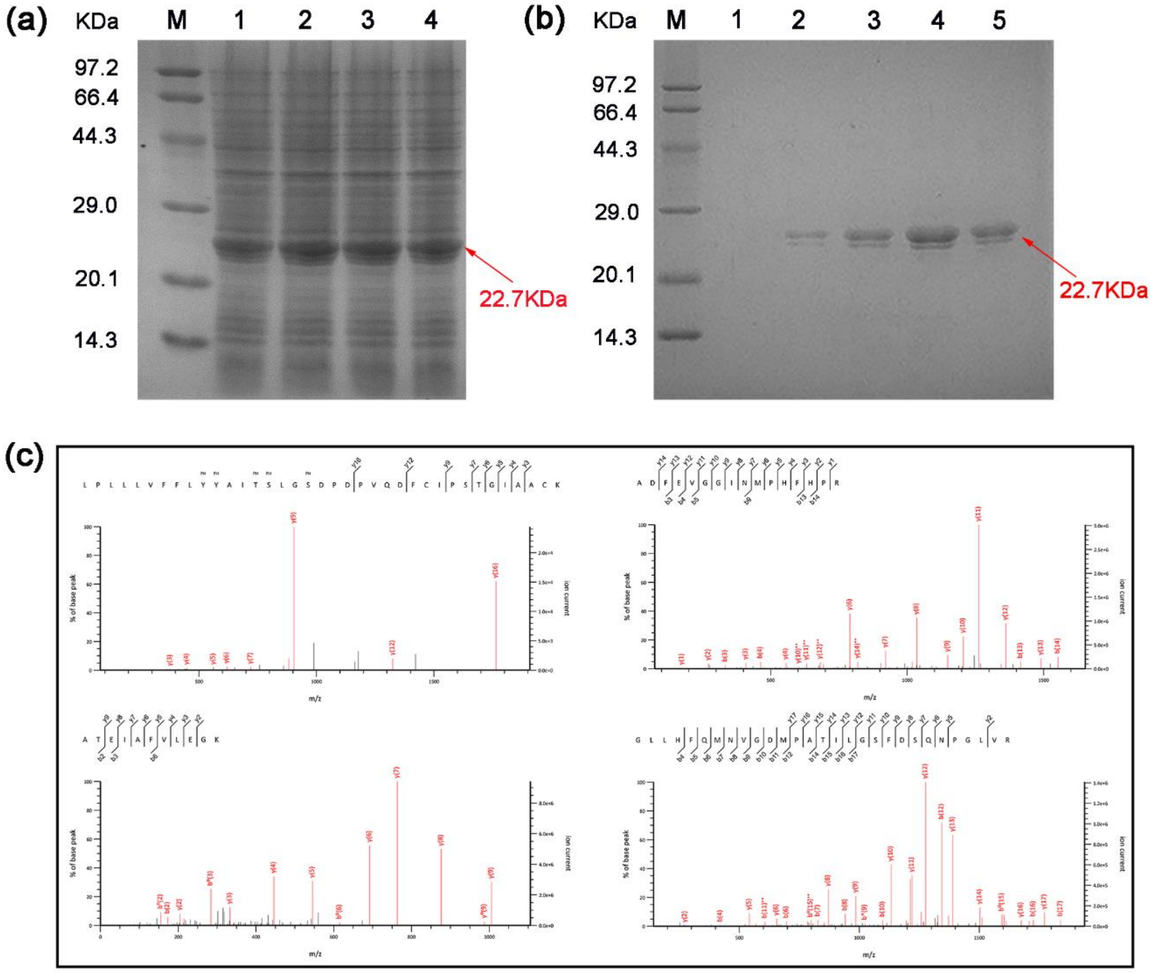

Supplementary Figure S1. Production and identify of GhGLP2 protein. **(a, b)** Production and purification of recombinant GhGLP2 proteins by Coomassie-stained SDS-PAGE. Expressed proteins are indicated in arrows. Lane M, protein molecular weight markers; **(a)** Lines 1-4, crude extract with 0.1 mM isopropyl-beta-D-thiogalactopyranoside induction, and cultured at 37 °C with oscillation for 2, 3, 4, and 5 h, respectively; **(b)** Lines 1-5, purified protein with different elution times. **(c)** MALDI-TOF mass spectra of the GhGLP2. The obtained recombinant protein spots were digested by trypsin, and the resulting peptides were analyzed by Mascot search.

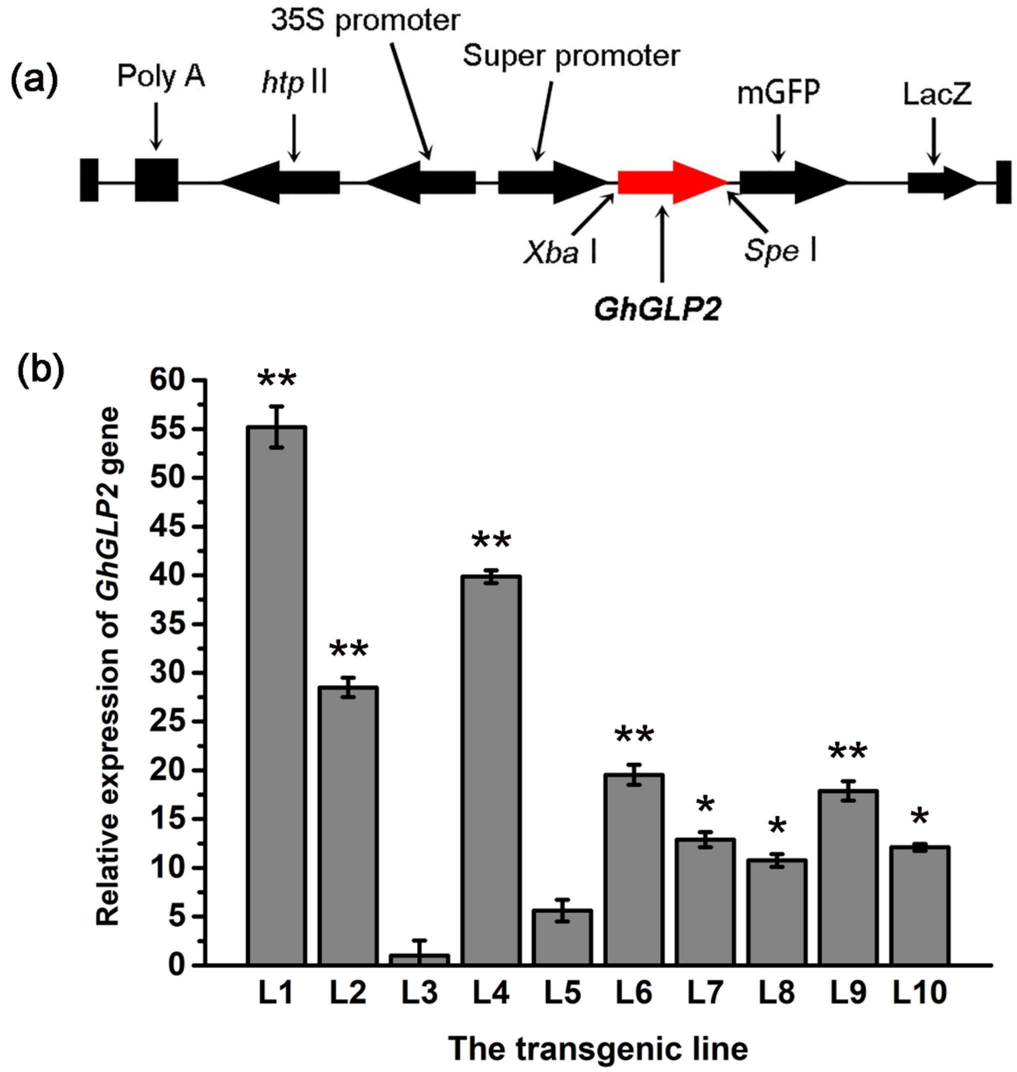

Supplementary Figure S2. Genetic transformation of *Arabidposis* with *GhGLP2* and identification of transgenic lines. **(a)** Outline of the Super-pCAMBIA1300 transformation vector with *GhGLP2* under the control of CaMV 35S promoter. **(b)** qRT-PCR analysis. Transcript levels of *GhGLP2* in transgenic *Arabidopsis* lines relative to the line with the lowest transgene expression (L3). All measurements were normalized with the *AtEF1a* transcript level. Data were collected from three independent biological samples per treatment and three technical replicates per samples. Error bars represent standard error. Asterisks indicate a significant difference ($^{*}P < 0.05$, $^{**}P < 0.01$, Student's t test).

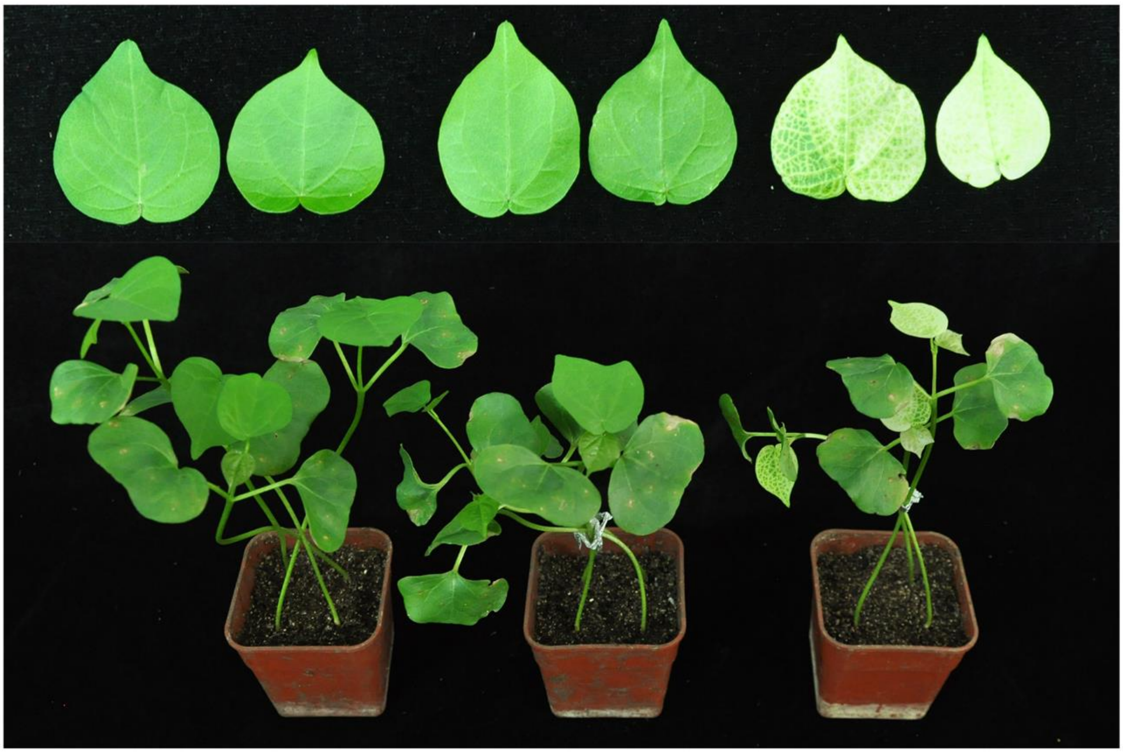

TRV:00 ***TRV:GhGLP2*** ***TRV:GhCLA1***

Supplementary Figure S3. The phenotypes of *TRV:00*, *TRV:GhGLP2* and *TRV:GhCLA1* cotton plants. The top panel shows the leaf phenotypes, the bottom panel shows the plant phenotypes.

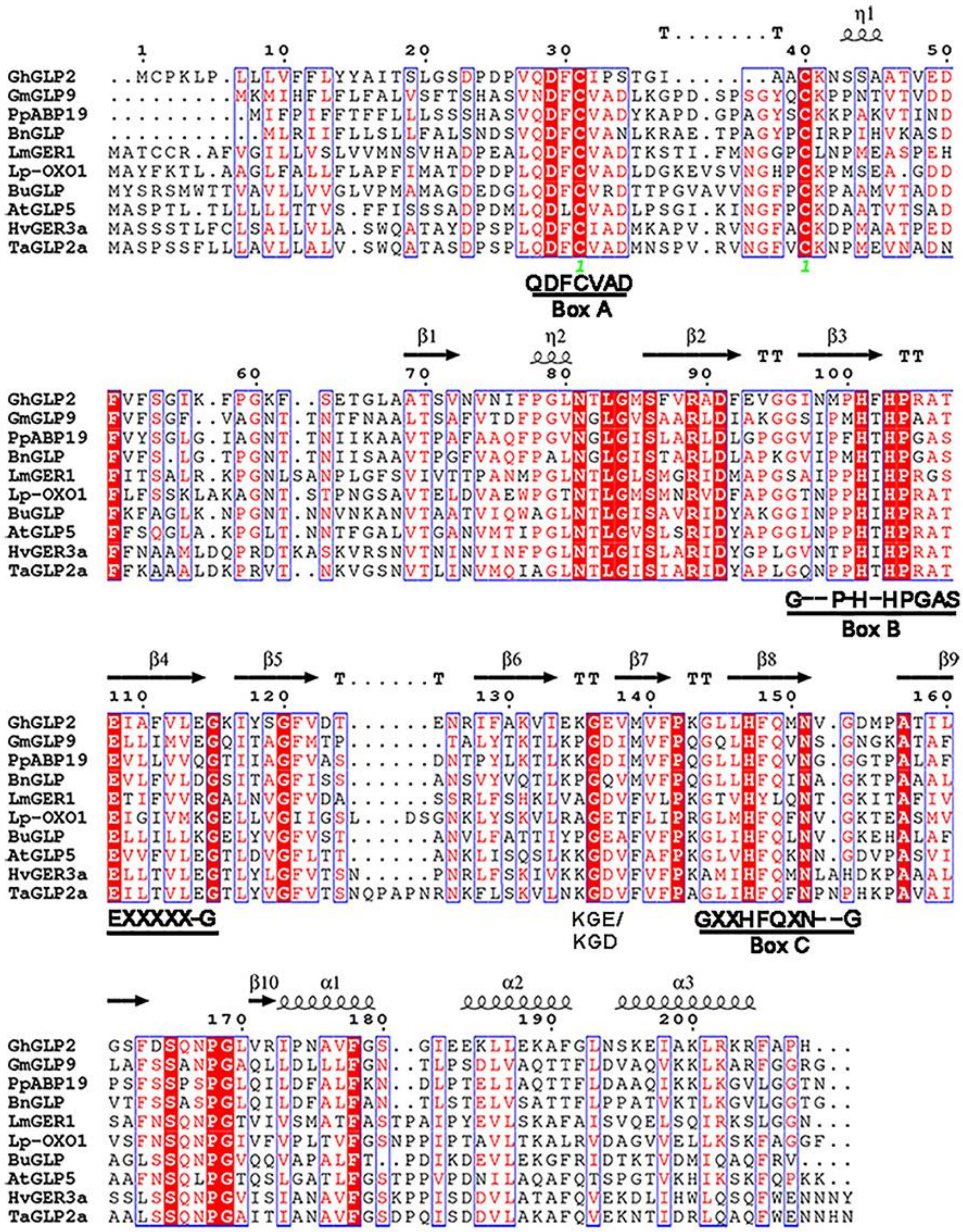

Supplementary Figure S4. Characterization and sequence analysis of GhGLP2. Amino acid sequence alignment of GhGLP2 and germin-like proteins from different plants. The conserved germin motif, box A, B and C are indicated in red (bolded) at the bottom, in which X corresponds to any hydrophobic amino acid residue. The alignment was performed using Clustal Omega and drawn using ESPript 3.0; β 1-10 indicate the jellyroll β-barrel domain of GhGLP2; α1, α2 and α3 in C-terminal indicate the three-helix bundle; the two conserved Cys residues marked with 1 at the bottom (in green) form one disulfide bond. Similar residues are colored in red and boxed, the invariant residues are red shadowed.

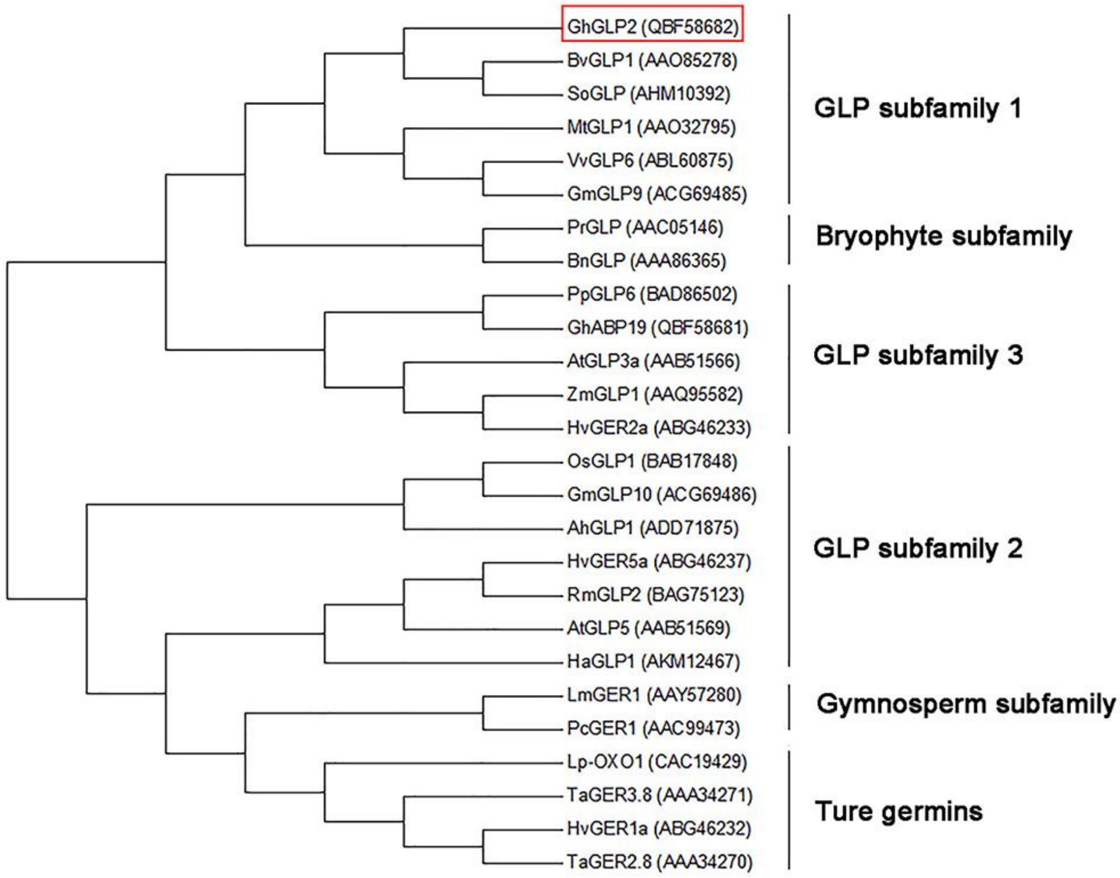

Supplementary Figure S5. Phylogenetic analysis of GhGLP2 together with other germin-like proteins. Multiple-sequence alignments were performed with neighbor-joining method by MEGA 7.0. The GenBank accession numbers are listed next to gene names; GhGLP2 from this study is framed by red boxes.

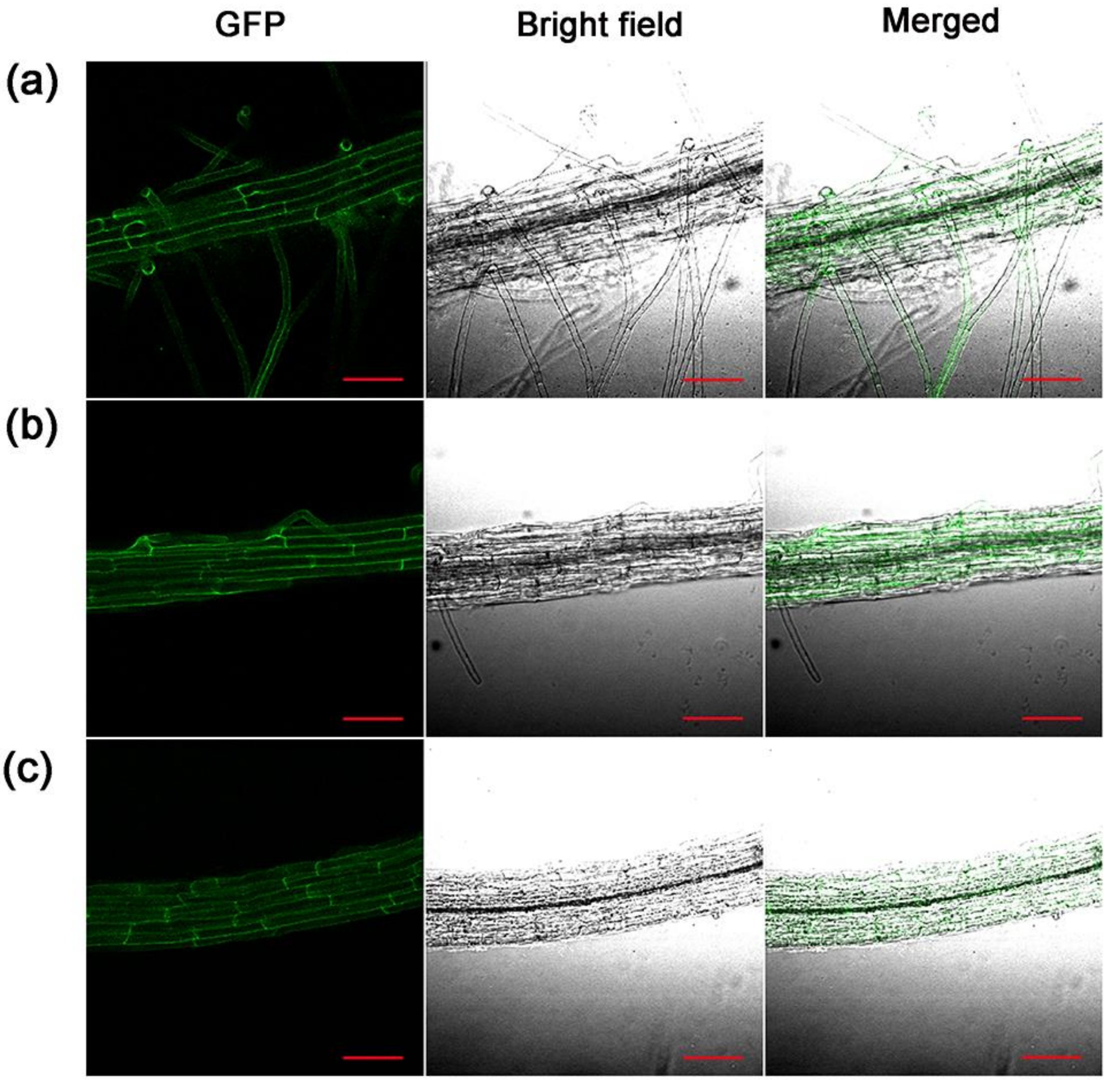

Supplementary Figure S6. Subcellular localization of GhGLP2:GFP protein in *Arabidopsis* root cells. **(a)** Wild-type *Arabidopsis*. **(b)** GhGLP2:GFP protein. **(c)** Plasmolyzed GhGLP2:GFP protein. Plasmolysis was induced with 0.8 M mannitol for 10 min. Scale bar represents 50 μm.

Supplementary Table S1. Sequences primers and restriction sites.

Primer name	Forward primer (5'-3')	Reverse primer (5'-3')
GhGLP2	CTAGTCTAGAATGTGTCCAAAGCTACCAC (*Xba* I)	AAGGACTAGTTGGAGCAAACCTCTTCCTC (*Spe* I)
pET-22b-GhGLP2	GGACCATATGTCTGACCCTGATCCTGTCCA (*Nde* I)	ACGCGTCGACATGTGGAGCAAACCTCTTCC (*Sal* I)
VIGS-GhGLP2	CGACGACAAGACCGTGACCATGGGCATAAAG TTTCCGGGGAAATT	GAGGAGAAGAGCCGTCATTACTTGGCAATC TCCTTGGAGTTCAA
qAtEF1α	ATTGGTAACGGTTACGCCCC	TCTCCTTACCAGAACGCCTGTC
qAtPR1	ACAACTAAGAGGCAACTGCAG	CCTTCTCGCTAACCCACAT
qAtPR2	CTACAGAGATGGTGTCA	AGCTGAAGTAAGGGTAG
qAtPR5	GTCTCGCCTCCAGTCAAACG	TTCAAGCCCTGCTCCAGAAA
qAtPDF1.2	TGCAAGATCCATGTCGTGCT	CAGAAGTTGTGCGAGAGGC
qAtLOX2	AACAGGCGACCCTAGTGATG	GCTCATTGGCCCAAGATGC
qAtAOS2	CAATACGTGTACTGGTCGAATGG	AAGGTGTCGTACCGGAGGAA
qAtVSP1	AACTGCAGAAGCTGGCACCTT	GTACTGGTCGTGGTTAGAGT
qAtCSD1	CCAAAGAGAGACGAAGCA	GCCTTTGTGATCTCAGGA
qAtCSD2	GCTCCAGAAGTGAGTGCCGTCA	CCACCCTTTCCGAGGTCATCCT
qAtRbohD	CGTACACACTCGTGCAATAATTGTG	TTCGAGTGGTTCAAGGAATAATG
qAtRbohF	GTTCGTTTCATCGTCGGAG	TTCGAGTGGTTCTTCGTAGCG
qGhGLP2	CCACGGTCGAAGATTTCGTG	CGGTATCGACGAATCCTGAG
qGhUBQ7	GACCCTTCCTCTATATAAG	GGACAACTCCATGAAAAG
qGhPR1	GGGGCAGTGCTGACCTATCG	TTAGCACAACCAAGATGGACAGAGT
qGhPR2	CTGGGCTTGTGGTCCTGGGA	CTTCCGTGACTTGGGTCAGT
qGhPR5	AAGGAGTCCACCACAATCACCG	CTGGCACTGCTATGGCTCGTAT
qGhPDF1.2	CTGTGGTAGCGGATGGTGATAAG	GTGCAGACGCATTTGCGAAGGAA
qGhLOX1	GCTTATGTTGCTGTAAATGACTCTGG	CACACTAAGTTGTCGGTTCGTTG
qGhVSP1	TTGTTCGATGAGTGGGTAATGG	CCTGAATACTTTGATGGTTCCTTG
qGhCSD1	AACTACCGTGACTGGGAACC	CGGGATCTGCATGGACAACT
qGhCSD2	GCTGTTACCAAGAAAGCTGT	TGCCACTCCATCAGCATTAG
qGhRbohD	CAACCATTCAGAAACAGGCC	AACAATGCCGAGCCACAACA
qGhRbohF	CACAAAGGTAAAGCTCCACG	CAGGAGCTATTATCACCACC
